# Nursing students’ perception towards educational environment in governmental Universities of Southwest Ethiopia: A qualitative study

**DOI:** 10.1371/journal.pone.0263169

**Published:** 2022-03-03

**Authors:** Melese Workneh Fego, Adugna Olani, Temamen Tesfaye

**Affiliations:** 1 Department of Nursing, College of Health Sciences, Mettu University, Mettu, Ethiopia; 2 Department of Nursing, Institute of Health Sciences, Wollega University, Nekemte, Ethiopia; 3 School of Nursing and Midwifery, Institute of Health, Jimma University, Jimma, Ethiopia; Murcia University, Spain, SPAIN

## Abstract

**Background:**

Educational environment refers to the diverse physical facilities, the clinical settings where students learn, the design and delivery of the curriculum, and involve the skills, and attitudes of the teachers. Due to various undesirable aspects of the educational environment, students often do not attain the expected professional standard of nursing care. There is limited evidence on students’ perception of their educational environment in Ethiopia, particularly in the study area. Thus, this study aimed to assess nursing students’ perceptions of their educational environment in government Universities of Southwest Ethiopia.

**Methods:**

An institution-based qualitative case study was employed. Eight participants were interviewed from government Universities of southwest Ethiopia. Face to face in-depth interview was used to generate data. The interviews were conducted for forty-five minutes to one hour using an open-ended semi-structured guiding questionnaire. All interviews were recorded, transcribed, translated, and analyzed using a conventional content analysis approach; finally, themes were derived and presented with narration.

**Results:**

The analysis of this study yields four themes categorized as curriculum, instructors’, organizations’, and students. The result of the study revealed that there were poor institutions students support systems, inadequate skills and knowledge in certain instructors, inadequate instructional materials and teaching aids, scarce facilities in students’ practical areas, accommodation, and transportation.

**Conclusion:**

Improvement of nursing students’ educational environment was recognized as a strong attention-seeking area. The majority of the key informants stated that the educational environment is not suitable for the teaching-learning process. Thus, to create a conducive educational environment, the Minister of education, Universities, instructors, and other concerned bodies should design and implement strategies targeted at the aforementioned problems.

## 1. Background

The educational environment (EE) refers to everything that surrounds an educational institution that represents the climate within the classrooms, department, and even the institution in general [1]. It contains learners’ viewpoints about infrastructures, learning opportunities, faculty skills, attitudes, and their peer’s socialization [1–5]. It has a crucial role in enhancing students’ growth, competency, critical thinking, independence, sense of mental wellbeing, and self-confidence [3]. Students’ satisfaction, academic success, and effective curriculum and instructors are the major indicators of teaching and learning quality and are related to several outcomes [4–6]. Besides EE influences how, why, and what students learn, which is vital in the success of the educational program [7].

A qualitative study conducted in Canada showed that confidence and competencies of educators, nursing students’ clinical experiences, subjectively evaluating the students’, poorly qualified instructors who lacked knowledge, skill, and teaching experience, poor relationships among instructors, staff nurses, and students’ were reported as the main challenges in nursing students’ in the clinical setting [8, 9]. A similar study done at Iranian University in 2015 showed that students’ perception about their theoretical education and clinical skills needs careful attention to the selection and recruitment of practical instructors by controlling their educational performances and clinical skills, and availing teaching equipment and facilities. It also revealed that the knowledge of instructors was not up to date and unskilled instructors were assigned for practical courses [10, 14].

Another qualitative study from a Norwegian University showed a shortage of teaching and learning tools, infrastructures, facilities, equipment, and standard procedures. The study also showed that expectations, feedback, and relationships as well as poor resources in the faculty and organization of the curriculum were known as factors that affect the students’ perception of their clinical skill learning environment [11].

In Sub-Saharan Africa, more reforms are needed to increase the capacity of educators and mentors responsiveness of curricula, strong regulatory frameworks, and availability of infrastructures and resources. Universities are responsible for the dissemination and transferring of knowledge, and providing specialized human resources [12, 13].

In Ethiopia, the role of higher education is the backbone for the country’s development efforts towards eradication of poverty and improving the vision of quality and employability of the university graduates. Thus, changing attention to quality, lead to change the schools into honest learning environments like quality-focused school supervision, internal school governance, increased students’ involvement, and school-community partnerships are among the priority focus [14]. There may be cultural and other determinants of how individuals view different qualitative aspects of a given EE, but perceived ratings report their perceptions [15].

The educational climate in nursing schools affects the quality and effectiveness of education [16]. Many qualitative studies have shown that there is a discrepancy between the views of students for class lectures and clinical attachments. It also appears unsupportive for their learning, mostly because of their teachers’ behavior [17]. The challenges and solutions in nursing education are common within the countries. There is a recognized association between positive EE and the development of learning capabilities of students’, performance and satisfaction [16, 18].

The goals of nursing education are mainly to equip nursing students with the required knowledge, attitude, skills, and professional ethics that will help them to solve health and health-related problems of the communities. Students’ comments’ are very important for the success of the educational environment [19]. Teachers play a vital role in promoting any educational system. Learning in the clinical teaching environment is considered to be central to the nursing education curriculum and for enabling nursing students to integrate their theoretical education into clinical practices. Therefore EE must be standardized to help nursing students’ to realize this combination [20]. While many studies were conducted in many countries in the world, currently little is known about nursing students’ perception towards their EE in Ethiopia. Thus, the present study was conducted to explore nursing students’ perception towards their EE in governmental Universities of southwest Ethiopia.

## 2. Materials and methods

An institution-based qualitative case study design was employed for this study seeks to provide a rich understanding of the study phenomenon contextually by researchers work. It is a better approach to understand the deep insight into the phenomenon of interest in the context of the case. In-depth investigation of small number of units at a point in time were done. Accordingly, a total of 8 participants(4 males and 4 females) were selected from Southwest Government Universities (two from Jimma University, two from Mizan Tepi University, two from Welkitie University and two from Mettu University) were selected purposively. Twelve class representatives from each university were contacted through the representative of students union and selected as key informants assuming that they had adequate information about the educational environment because of their closeness to university and department affairs. Using a convenient sampling technique, the sample size were determined by data saturation. Also we had considered resource constraints. All of the participants were year four students, and age groups between 21 to 33. Data was collected by four experienced interviewers from April 9 to 23/2019 through face-to-face in-depth interviews in the silent and private rooms. A semi-structured interview guide, digital recorder, and field note were used to capture interview data. An initial probe during the first interview was primarily directed to the respondent to expand or explain some of his/ her answers as appropriate to the flow of the interview. The time needed for each interview session was between forty-five minutes and an hour.

Interview data were transcribed and translated from local languages to English verbatim on daily basis, to guide the subsequent interviews. Field notes were summarized daily in English to prepare for coding. Data were analyzed using the conventional content analysis approach. Accordingly, to identify the topics, codification of the text, emerge codes, and creation of the categories, each text was read again and again carefully to advance a common understanding of the vital statements. Then, meaning units about participants’ experiences of nursing education, existing problems, and challenges were extracted from the transcriptions. The meaning units were abstracted by summarizing the vital statements’ and labeled as codes. The respondents’ speeches and secret concepts were used for coding, and then the codes were compared for matches and differences. Accordingly, the classification of codes was carried out. Finally, four themes were emerged based on the results. Experts were also asked to review the themes to reach a mutual agreement on related codes and the classifications of the data. Finally, the interview result was written by compiling memos, quotes, and analyzed data, and presented with narrative descriptions.

The ethical clearance with reference number GHRPGD/501/2019 was obtained from the Institutional Review Board (IRB) of Jimma University, Institute of Health, and submitted to the concerned bodies. Informed verbal consent was obtained for the digital recording of an interview before beginning any formal interview. The Code of participants was used for all participants in data records (field notes, interview transcripts, and translated data). The transcripts were devoid of real names or places thereby ensuring the complete anonymity of the participants. Hard data (printed, translated, transcripts, and field notes) were stored in a private room. Electronic versions of these documents were stored on password-protected computers.

## 3. Results

The perception of the educational environment was explored by the experience of the respondents. The main themes that emerged from the qualitative analysis of nursing students’ perception of their educational environment were related to curriculum, instructors, organizations, and students’ (S1 Table). The results for various categories within the themes were presented and illustrate the findings with citations from the interviews.

### 3.1 Curriculum

#### 3.1.1 Curriculum contents

This theme was directly extracted from curriculum contents and its application strategies. Many respondents were perceived that the working nursing curriculum contents are not sufficient to address the need of the nursing profession and not to the standard. They also perceived that the course delivery system was not convenient for students to acquire the required knowledge and skills. A participant 1 said that: “…the curriculum contents are not comprehensive, redundant and contain courses not related to a nursing profession like civic…; ….the given time for theory is higher than practices” (Participant 4). The other participant reported that: “…in block courses delivery system, we did not remember what we have learned after the exam, but in the semester-based system, we remember the subject matter very well even after a long time” (Participant 5), (S1 Table).

#### 3.1.2 Curriculum implementation strategies

The students’ described that even though the curriculum implementation strategies were good; there was a lack of monitoring and evaluation strategies for curriculum implementation. One study participant supports this idea by saying: “…there was no regular supervision by the departments, faculties/colleges or other concerned bodies on curriculum implementation…” (Participant2). Many participants recommended the need for curriculum revision related to the course delivery system, theory-practice balance, and avoid the redundancy of contents. Other participant revealed that: “…the nursing curriculum should be revised… course delivery system for all courses should be semester-based…; the given time for practical attachment should be more than theory class…to create well conducive learning environments” (Participant 6). Further participants recommended that: “…better to take ethics courses at first year, research methodology at the last year; also like health officer and medicine students’, nursing students’ should stay at the campus during the summer season” (Participant 4), (S1 Table).

### 3.2 Instructors’

This theme was directly extracted from teaching methods, evaluation methods, skills, and knowledge and behavior of instructors. Most of the students’ opined that some instructors were not adequately prepared for the classroom and clinical teaching. Moreover, they were dependent on single teaching methods and instructional media. Participant 3 said that: “…Some instructors used only one way of teaching methods…. and they depend on a limited type of instructional materials and they did not use other alternative instructional methods, as a result, they omit classes during electric power interruption”. Also, participant 8 said: “…most instructors’ did not follow the adult learning approach and they did not give course outlines on time”. Concerning the evaluation of students’, the participants expressed that the instructors did not prepare the exams in line with the course objectives. Very often the questions for the exams were downloaded from the internet. Few instructors was evaluated their students’ based on social intimacy. Participant 2 said that: “…Some instructors did not evaluate students fairly and they provide grade with intimacy; … the evaluations were without the course objectives, test and exam questions are full of subjectivity and out of the learning objectives and discussion in the classes” (Participant 2). The other respondents reported that: “…a small number of instructors were preparing the exam from the internet which not focuses on the topics of subject matter and there was poor remedial action system for under scorer students” (Participant 7). The participants recommended that the instructors should use multiple teaching aids, follow the pedagogical learning approaches, prepare themselves very well for teaching, should be fair in grading regardless of intimacy and any other conditions, evaluate based on the learning objectives and avoid preparing the test or exams from internet, (S1 Table).

Regarding the knowledge and skills of instructors, almost all of the participants reported that very few instructors had poor teaching experiences, lack of proper skills in practice, and most instructors were also not well prepared for class teaching. One participant revealed that: “…there was a gap between educational need with teaching skills and knowledge in very few instructors” (Participant 1). Students’ described the instructor’s behaviors as lacking punctuality, not interested in showing exam results, an unethical approach to students’ and having a dictatorial attitude. Other participant said that: “…many instructors did not respect the time; some of them didn’t want to show test or exam results; few instructors’ were authoritarian or dictators” (Participant 8). Another student’ also reported that: “…Some instructors were unethically intimate with students’, and most instructors are unethically acting during a teaching in the classrooms” (Participant 5), (S1 Table).

### 3.3 Organizations’

This theme was directly extracted from teaching and learning facilities, infrastructures, and students’ support systems. Interviewees described a shortage of instructional materials, skill lab equipment, and equipment for practicing in the clinical area from all universities. A Participant 1 reported that: “…there were inadequate instructional materials or teaching aids in the university, for instance, Liquid Crystal Display(LCD), skill development lab equipment’s, shortage of clinical learning equipment like blood pressure apparatus, thermometer, personal protective equipment’s, projectors, photocopy machine, dividers, digital library or computers, reading materials, whiteboard markers, different audiovisuals, flip charts…. and some of the available materials were also not functional”, (S1 Table).

Regarding infrastructure, the majority of the participants were described as a lack of a favorable learning environment due to a lack of well-established infrastructures. One respondent explained that: “…there were frequent interruptions of electric power and water supply, poor internet accesses, inadequate number of lecture halls, skill development laboratories, tutorial classes, peer learning classes” (Participant 3). The other respondent also supplemented that: “…lack of electric power in most buildings, lack of dormitory inside the hospitals during practices” (Participant 4). Noise and dust, blocking of routes, traffic, etc… arising from different constructions within the University’s campus contributed to an unfavorable and disturbing educational environment. Participant 7 said: “…there were poor internal roads which were unfavorable to move around to use basic services and road under construction made the environment not conducive for teaching and learning due to noises”.

Regarding the students’ support system, the study participants feel that Universities had a poor supporting system for those in need. Respondent eight revealed that: “…there was an inadequate support system for the poor students’, shortage of medications in students’ clinic, inadequate support system during clinical practice like transportation, houses or dormitory, tuition fee payment processes”, (S1 Table).

### 3.4 Students’

This theme was extracted directly from learning motivation, students’ behavior, academic performance, and students’ satisfaction. Most respondents admitted that many students lacked the motivation to learn. Because of this some of them had no desire to read and prepare themselves. Students’ lacked discipline in the classroom by talking and using cell phones during the lecture. Other Participant stated that: “students’ did not obey in the classroom, they are also not adequately interested to learn new things from their instructors, and they had poor habits for study” (Participant 6). The other respondent also revealed that: “Very few students’ were misbehaving in the class and turn, they disturb teachers during teaching and learning process, few students’ were using substances, improper time utilization (playing games, waste time on social media, watching films), don’t want to study in the library and with their peer learning groups” (Participant 4).

Regarding students’ academic performance, the majority of the participants reported that the higher scorer students’ have a positive perception of their educational environment and vice versa. Participant 3 reported that: “students’ perception relies on teachers grading without their input”. Regarding Students’ satisfaction, the majority of the participants described that Students’ satisfaction was highly dependent on high current cumulative grade point average (CCGPA) scores and their support systems. Thus, they recommended that students should respect their profession and read well. They also suggested that the Universities should support stressed students and make teaching and learning processes more conducive, (Participant 3), (S1 Table).

## 4. Discussion

The educational environment is the cornerstone for the better productivity of higher educational institutions. A less conducive educational environment reduces the performance of students and a reason for the poor quality of education. Currently, throughout the world, there is limited information about nursing students’ perception of their educational environment [6, 7]. Thus the present study explored nursing students’ opinions towards their educational environment in governmental Universities of southwest Ethiopia (S1 Table).

This study showed that the contents of the current nursing curriculum inadequately addressed the need of the profession and it is not to the standard. Some courses are redundant and unrelated to the nursing profession like civics. The participants believed that hours given for the theory were more than the hours allotted for clinical practice. Besides, the study showed a lack of uniformity with block and semester-based delivery of courses. The curriculum was haphazardly implemented during a block-based system. Thus, the participants recommended shifting all the block courses to the semester-based delivery system and the pressing need for nursing curriculum revision. This study had similar findings with studies conducted at the Kuate University of Saud Arabia, a Private University of Malaysia, and a qualitative study conducted in Iran [5, 10, 11].

The current study also revealed that students felt nursing instructors had inadequate knowledge and skills to teach nursing students, particularly in the clinical area. The majority of respondents reported that some instructors are unethical, biased in students’ evaluation, preparing examinations out of the learning objectives especially from the internet, and had not respected their students’. The result also showed that most instructors were using one-way teaching methods; merely relied on projectors or LCD and they did not use alternative teaching aids in the event of power failure. Moreover, participants also reported that some instructors are authoritarian; they did not follow the course syllabus or give reading materials on time, or show the students’ assessment results. The result of this study was different from a study conducted in three Countries including Aga khan university medical college of Karachi in Sweden, Pakistan in Shifa College of Nursing, and Addis Ababa University, Ethiopia [4, 12, 17]. The possible explanation of the discrepancies might be due to differences in the level of instructors teaching knowledge and skills, behaviors, and characteristics. Also, the difference might be due to a lack of close follow-up and supportive supervision from concerned bodies for those instructors who had inadequate knowledge and skill and harsh behavior.

The result also exposed the lack of teaching and learning facilities/instructional materials like smart classes, desktop computers, LCD, learning videos, manuals, whiteboard markers, and flip charts. Also, the study brought to light poor infrastructure in the study areas like poor internet access, inadequate lecture halls, tutorial classes, and an insufficient support system for needy students’. Lack of support was described as inadequate medications in the clinic, poor transportation, and lack of support system in the hospital practices. These findings are contrary to the study conducted in four Countries namely, University College of Southeast Norway, Students’ perception of the educational environment of medical schools in Korea, Iran, and Addis Ababa University, Ethiopia [5, 7, 8, 17]. The discrepancy might be due to differences like medical students’ perceptions of their educational environments compared to nursing students’. It might also be due to the nursing profession is more reliant on skills that demand many skill learning equipment and materials that expose them to a higher expectation. Moreover, the availability of well-established infrastructures and teaching-learning materials are directly associated with the development of countries and regions. Thus, in a developed country like Korea, these kinds of problems may not exist.

The result of the present study showed that lack of motivation to learn from many students’, unethical behaviors in some students, low academic performance, and disinterest in the profession. The participants recommended that students’ should be motivated for learning new things, studying hard to attain high grades and love, and respect their profession. These results are in line with many studies conducted in different countries in the world [4–10, 13, 17, 21].

### 4.1 Limitation

The limitations of this study were lacks representativeness for similar populations due to the economic and cultural differences, and differences in Nursing programs that might be affecting the transferability of the result.

## 5. Conclusion

Even though the participants were optimistic about their educational environment, most of them rated it as not conducive for teaching and learning. The study also revealed that there was poor implementation practice of the curriculum, poor institutions students support system, inadequate skill, and knowledge in some instructors, scarce instructional materials or teaching aids and infrastructures, scarcity of equipment’s for practicing in skill development lab and clinical settings, and lack of motivation from the students’. Thus, Universities should design follow-up and supportive supervision mechanisms for instructors who have knowledge and skill gap and misbehaving towards their students’. In addition monitoring and evaluation of curriculum implementation practices, enhancing the University infrastructures and instructional materials, creating conducive practical areas, improving the motivation of students’ and about adequate students’ support system are important. Also, the Minister of Science and Higher Education of Ethiopia should consider curriculum revision from a block-based to a semester-based approach.

## Supporting information

S1 Table Themes emerged, categories, and codes of nursing students’ in governmental Universities of southwest Ethiopia, (n = eight). (PDF)
