# Supplementary material for: Nursing students’ perception towards educational environment in governmental Universities of Southwest Ethiopia: A qualitative study
Source: PLoS One. 2022 Mar 3;17(3):e0263169. doi: 10.1371/journal.pone.0263169 (PMC8893652; doi:10.1371/journal.pone.0263169)
Supplement: S1 Table — (PDF) [file pone.0263169.s001.pdf]

Supporting Information

Table 1: Themes emerged, categories, and codes of nursing students' in governmental Universities of southwest Ethiopia, (n=eight)

Meaning units	Condensed meaning Units	Sub-category	Categories	Themes
-Curriculum contents and given time are unsuitable	-The curriculum contents, given time, and consistency are inappropriate	-Topic contents and time mismatch<br>-The given for practice lower than theory<br>-Lack of uniformity (block and modular courses.<br>-Full of redundancy in some topics<br>-Unsuitable contents like civics, sociology, psychology, etc…	Curriculum contents	**Curriculum**
-The curriculum did not constantly applicable	-Lack of regular application of the curriculum	-Poor follow up in many teachers<br>-Not well implemented.<br>-The syllabus did not offer at the start.<br>- Contents not well applicable<br>-Inadequate follow-up	Application strategies	
-Traditional teaching methods	-Low chances for students' to ask questions	-One-way teaching methods<br>-Using a few instructional materials<br>- Weak students' participation<br>-Not adult learning approach<br>-Not providing course outlines on time	Teaching methods	**Instructors'**

-Un-ethical and unfair evaluation methods	- Most teachers did not allow to see test and exam results<br>-Under scorers not allowed to take re-exam on time<br>-Exam contents out of the given materials	- Lack of fairness in grading (grading with intimacy)<br>-Evaluation without curriculum<br>-Subjective questions<br>-Exam out of subject matter<br>-Lack of remedial action for below-average scorer students'.<br>-Exam from the internet	Evaluation methods	
-Poor teaching skills	-Poor teaching skills, preparation, educational degrees	-Poor teaching experiences<br>-Lack of suitable skill in practice<br>-Lack of grasping subjects<br>-Poor preparation for teaching<br>-Mismatch of educational degree and teaching skills and knowledge	Knowledge and skills	
-Un-ethical act and dictators	-Un-ethicality during communication, lecturing, and testing or evaluation.	-Lack of respecting lecture time<br>-Don't want to show the test result<br>- Authoritarian teachers<br>-Unethical intimacy with students'<br>-Unethical act in teaching	Instructors behavior	
Scarcity of teaching aids, lab and practical	-Inadequate teaching materials for lecturing, skill lab, and practices	-Scarcity of teaching aids (skill lab equipment and materials, LCD, copy machine, dividers, Computers, reading materials, etc…	Teaching and learning facilities	**Institutions'**

equipment's		-Available teaching aids not functional<br>-Lack of clinical learning equipment's<br>- Lack of papers, photocopy machine for exam		
-Scarce of infrastructural facilities.	-Inadequate infrastructural facilities like electric power, water, internet, lecture classes, roads, etc…	-Frequent interruption of electric power<br>-Scarcity of lecture classes, skill lab room, etc…<br>-Lack of electric power in most buildings<br>-Lack of dorm in the hospitals during practice<br>-Lack of road and road under construction<br>- Lecture class wall was not clean<br>-Improper buildings	Infrastructures	
Weak support system for stressed students'	-Weak attention for poor students' and scarcity of medications	-Inadequate support for the poor students'.<br>-Shortage of medications in students' clinic.<br>-Inadequate support in clinical practice (transport, house, etc...)	Students' support system	
-Demotivated students'	-Lack of motivation for lectures, hospital practices, and study	-Did not adhere to the learning process<br>-Low inspiration to learn new things from teachers<br>-A poor habit of regular study	Learning motivation	**Students'**
-Un-ethical, time-wasting, dependent on substances to study	-Un-ethical act in the class, wasting time to study, using substances for study	-Using substances for study<br>-Improper time utilization(playing games, Facebook, watching films)<br>-Don't want to study in the library and with a	Students' behavior	

		group<br>-Stop to study during underscoring.		
-Perception relies on their scores	-Perception reduced with low scores	-Perception varies with scores or CCGPA<br>-Many students' weaker in their academic status	Academic performance	
-Perception affected by students' satisfaction	-Dissatisfied with a weak support system and with lower CCGPA	-The satisfaction due to high CCGPA raise perception<br>-Satisfaction in the support system, teaching and learning processes, profession, etc.	Students' satisfaction	

Key: Theme: is a higher-level of categorization, we used to identify a major element of our entire content analysis of the text. **Themes'** are the experiences of the participants that the researcher sees as pertinent to the research question. The themes are identified by coding. **Coding'** is the process of identifying themes in explanations and attaching labels. **Category**: is grouping the coded segments, to reduce the number of different pieces of data in our analysis. It also a collection of similar data sorted into the same place and enabled us to identify and describe the features of the category. **The sub-category** is possibly turning the initial category into a sub-category. **Meaning unit** in this study is the operationalization of a research question that enabled us a certain kind of measurement. **The condensed meaning unit** is the interpretation of the underlying meanings.
